# A decorated raven bone from the Zaskalnaya VI (Kolosovskaya) Neanderthal site, Crimea

**DOI:** 10.1371/journal.pone.0173435

**Published:** 2017-03-29

**Authors:** Ana Majkić, Sarah Evans, Vadim Stepanchuk, Alexander Tsvelykh, Francesco d’Errico

**Affiliations:** 1 Centre National de la Recherche Scientifique, UMR 5199 - PACEA, Université de Bordeaux, Pessac, France; 2 Division of Archaeology, University of Cambridge, Cambridge, United Kingdom; 3 Institute of Archaeology of NASU, Kiev, Ukraine; 4 I.I. Schmalhausen Institute of Zoology of NASU, Kiev, Ukraine; 5 Evolutionary Studies Institute and DST-NRF Centre of Excellence in Palaeosciences, School of Geosciences, University of the Witwatersrand, Johannesburg, South Africa; Institucio Catalana de Recerca i Estudis Avancats, SPAIN

## Abstract

We analyze a radius bone fragment of a raven (*Corvus corax*) from Zaskalnaya VI rock shelter, Crimea. The object bears seven notches and comes from an archaeological level attributed to a Micoquian industry dated to between 38 and 43 cal kyr BP. Our study aims to examine the degree of regularity and intentionality of this set of notches through their technological and morphometric analysis, complemented by comparative experimental work. Microscopic analysis of the notches indicate that they were produced by the to-and-fro movement of a lithic cutting edge and that two notches were added to fill in the gap left between previously cut notches, probably to increase the visual consistency of the pattern. Multivariate analysis of morphometric data recorded on the archaeological notches and sets of notches cut by nine modern experimenters on radii of domestic turkeys shows that the variations recorded on the Zaskalnaya set are comparable to experimental sets made with the aim of producing similar, parallel, equidistant notches. Identification of the Weber Fraction, the constant that accounts for error in human perception, for equidistant notches cut on bone rods and its application to the Zaskalnaya set of notches and thirty-six sets of notches incised on seventeen Upper Palaeolithic bone objects from seven sites indicate that the Zaskalnaya set falls within the range of variation of regularly spaced experimental and Upper Palaeolithic sets of notches. This suggests that even if the production of the notches may have had a utilitarian reason the notches were made with the goal of producing a visually consistent pattern. This object represents the first instance of a bird bone from a Neanderthal site bearing modifications that cannot be explained as the result of butchery activities and for which a symbolic argument can be built on direct rather than circumstantial evidence.

## Introduction

Neanderthals’ cognitive abilities are a hotly debated topic. Opinions differ radically between those who think that Neanderthal cognition was comparable in many, if not all respects, to that of contemporaneous and present day Modern Humans [1–16], and those who believe that differences in cranial morphology [17–20], ontogenesis [21–24], physiology [25–27], and behaviour [28–42] support a different cognition. Paleogenetic data demonstrating a significant interbreeding between Neanderthals, Modern Humans, and Denisovans [43–47] show that differences in cognition, if any, did not prevent members of these populations from recognizing each other as desirable mates, and securing successful social integration and reproduction of hybrids. Such an outcome would arguably be unlikely in the face of major cognitive differences and if, for example, language was absent [13,48]. A growing body of archaeological discoveries and reappraisal of old finds is also strengthening the position of those who advocate comparable cognition. Complex and changing lithic technologies, hafting techniques, varied hunting strategies enabling Neanderthals to kill dangerous game and exploit a variety of marine [49,50] and plants resources [51–55], established ability to ignite and control fire [56], and organisation of living space, are among the innovations that are now recognized as inherent to Neanderthal cultures in various regions of Europe, before any contact with Modern Humans [4,8,9,14,57,58].

In addition, several lines of evidence—burials, collection of rare items, production of engraved and perforated objects, personal ornaments, pigment use, and the extraction of bird feathers and claws—support the notion that Neanderthals engaged in symbolically mediated behavior, independently from the influence of anatomically modern humans. Neanderthal burials of infants, children, and adults have been reported at several sites throughout Europe and the Near East, some of which are associated with grave goods [59–62] (but see [63] for a different view). Several Mousterian sites (Canalettes, Combe Grenal, Grotte de l’Hyène at Arcy, Tabatérie, Chez Pourré-Chez-Comte, Cioarei-Borosteni,) yielded evidence for the collection of rare objects in the form of crystal and fossils [64]. Pigment use among Neanderthals dates back as far as 200–250 ka [65], and becomes a more widespread practice after c. 60 ka, as testified not only by the finds of modified ochre, manganese and graphite pieces [14,58,64,66,67] but also processing tools and possible pigment containers [68–72]. An ochered fossil marine shell has been discovered in a Mousterian level dated to at least 47.6–45.0 cal kyr BP at Fumane cave in Italy [73], and ochered marine shells come from Cueva de Los Aviones and Cueva Antón archaeological layers dated to c. 50 ka, in the Iberian Peninsula [74].

Bone and stone objects bearing multiple incisions, interpreted as deliberate, possibly symbolic, engravings, are reported from more than forty European sites dated to the Lower and Middle Palaeolithic. A number of these incisions have been reinterpreted as the consequence of natural phenomena [75]. Many others wait for detailed analysis in order to verify the agent responsible for the modifications, and evaluate to what extent, when human made, the incisions may be better explained as the result of butchery or other subsistence activities, or as the expression of symbolically mediated behavior. A case in point is the recently discovered crisscross pattern deeply incised into the bedrock of Gorham`s Cave, Gibraltar, which represents the first reported instance of rock art produced by Neanderthals [76].

Even if it is becoming compelling, however, the evidence is spatially unbalanced with more, or more detailed and recently acquired data, only available for some regions and little or ambiguous information published for others. Another problem that researchers face when assessing instances of Neanderthal complex behavior is linked to the known difficulty of inferring cognition from material culture. Arguments in favor of equal cognition mostly rely on general comparison with the UP or the cultural adaptations of historically known hunter-gatherer populations. Attempts to reconstruct and evaluate past hominin cognition are either based on the analysis of cognitive processes at work among modern experimenters when performing activities similar to those conducted in the past [77–81] or from more general frames of inference, such as the *chaîne opératoire* concept [82–86], which try to broadly evaluate the cognitive implication of past behaviour or social transmission strategies [87–90] by inferring them from the detailed analysis of archaeological artifacts. In this study, devoted to bird bone decoration by Neanderthal, we will follow a novel research strategy that includes experimental work aimed to compare markings produced by modern humans under specific neuromotor constraints with archaeological notches. Recording the same variables for archaeological and experimental markings allowed to assess the degree of regularity and intentionality reflected by the former.

### Weber-Fechner law

Our visual brain demonstrates a universal and reliable range of capacities and limitations with which we can detect and perceive our environment. One such limitation is our capability to distinguish a difference in magnitude of a particular characteristic for two stimuli, such as the difference in length between two distances. The amount of change needed in one stimulus in order for it to be perceived as different from another is considered to be the difference threshold, or just noticeable difference (JND).

The Weber-Fechner law [91,92] states that this error in human perception is constant and proportional to the magnitude of the stimulus in question; this constant is termed the Weber Fraction. A different constant exists for a variety of characteristics, such as length, weight or taste, and provides the minimum difference detectable without an aid. For line length, or the distance between two points, the Weber Fraction has been determined to be 0.029 or 0.030 [93–95]. Thus, if one line or distance was larger by 3% or more than another, the difference in magnitude would be perceived, whereas a difference of less than 3% would result in the two distances being viewed as equal.

This approach, when adapted to the production and perception of notches, provides a quantitative measure for evaluating the regularity of spacing in notches, i.e. whether the distance between two notches is perceived to be the same as or different from another distance between two notches. The utility of this principle within an archaeological context has been recognised previously, particularly with reference to material standardisation and the amount of variation in object size [96–99]. Above all, the application of universal and reliable neurophysiological and psychophysical principles can serve as a link between our brains and those of our ancestors, and provides a quantitative method of assessing the production, manipulation and perception of archaeological artefacts.

Here we report on a bird bone from the Middle Palaeolithic site of Zaskalnaya VI (Kolosovskaya) Crimea, which bears a set of evenly spaced notches that cannot be explained as resulting from butchery activities. The technological analysis of these notches and their comparison with sets of notches produced by skillful experimenters or present on UP objects identify behavioral consistencies demonstrating the ability and intention of producing a visual conformity comparable to the one that characterizes modern human productions and reflects modern cognition.

### Neanderthal bird exploitation

A string of new discoveries, related to bird exploitation, has recently enlarged the panoply of activities conducted by Neanderthals that may reflect their involvement in symbolic activities (Table 1). Sixteen Mousterian and Châtelperronian sites from Italy (Fumane, Rio Secco), Gibraltar (Gorham’s Cave, Vanguard, Ibex), France (Baume de Gigny, La Ferrassie, Combe Grenal, Les Fieux, Mandrin, Grotte de L’Hyene, Grotte du Renne, Grotte du Noisetier, Pech de l’Aze I and IV), and Croatia (Krapina) have yielded terminal phalanges of seven bird species with cut-marks indicating that Neanderthals deliberately removed the claws [100–117]. At seven Mousterian sites from Italy (Fumane), France (Grotte du Noisettier, Lazaret, Le Fieux), Germany (Salzgitter-Lebenstedt), and Gibraltar (Vanguard and Gorham’s Cave) cut-marks and scraping marks on upper limb bones indicate that feathers were purposely detached from the wings. Removal of feathers and claws is interpreted as proof that these objects were used as personal ornaments by Neanderthals. Since feathers and claws do not survive archaeologically and no clear modifications for suspending or threading the claws were found so far on bird talons, this hypothesis exclusively lies on circumstantial evidence, i.e. evidence that relies on an inference to connect it to a conclusion. In archaeology, the weakness of hypotheses based on circumstantial evidence is that they do not escape the danger of equifinality, and are difficult to test.

**Table 1:** Mousterian and Châtelperronian sites with evidence of bird exploitation and modified bird bones.

						Taxon							Modification			Interpretation			
Site	Country	SEMP	Layer	Age*	Cultural attr.	Order	Family	Scientific name	Common name	Undetermined	Skeletal element	NISP	Cut marks—NISP	Notches	Sawing	FE	TE	Other	Reference
Baume de Gigny	France	No	XV	50	MP	Anseriformes	Anatidae	*Cygnus cygnus*	Whooper swan	N/A	Pedal phalanx	1	1	-	-	-	X	-	[105,106]
Combe Grenal	France	Yes	52	90	MP-CM	Accipitriformes	Accipitridae	*Aquila chrysaetos*	Golden eagle	N/A	Pedal phalanx	1	1	-	-	-	X	-	[105]
Fumane	Italy	No	A12	MIS 3	MP-L	Accipitriformes	Accipitridae	*Aquila chrysaetos*	Golden eagle	N/A	Pedal phalanx	1	1	-	-	-	X	-	[101,102]
Fumane	Italy	No	A9	48–49	MP-D	Accipitriformes	Accipitridae	cf. *Gypaetus barbatus*	Bearded vulture	N/A	Ulna	1	1	-	-	X	-	-	[102]
Fumane	Italy	No	A9	48–49	MP-D	Accipitriformes	Accipitridae	*Aegypius monachus*	Cinereous vulture	N/A	Carpometacarpus	1	1	-	-	X	-	-	[100,101]
Fumane	Italy	No	A9	48–49	MP-D	Accipitriformes	Accipitridae	cf. *Aegypius monachus*	Cinereous vulture	N/A	Ulna	1	1	-	-	X	-	-	[101]
Fumane	Italy	No	A9	48–49	MP-D	Accipitriformes	Accipitridae	*Clanga clanga*	Greater Spotted Eagle	N/A	Radius	1	1	-	-	X	-	-	[101]
Fumane	Italy	No	A9	48–49	MP-D	Falconiformes	Falconidae	*Falco columbarius*	The merlin	N/A	Carpometacarpus	1	1	-	-	X	-	-	[101]
Fumane	Italy	No	A5-A6	40–45	MP-L	Accipitriformes	Accipitridae	*Gypaetus* cf. *barbatus*	Bearded vulture	N/A	Ulna	15	1	-	-	X	-	-	[100,105]
Fumane	Italy	No	A5-A6	40–45	MP-L	Falconiformes	Falconidae	*Falco vespertinus*	Red-footed Falcon	N/A	Humerus	1	1	-	-	X	-	-	[100]
Fumane	Italy	No	A5-A6	40–45	MP-L	Columbiformes	Columbidae	*Columba palumbus*	Common wood pigeon	N/A	Carpometacarpus	103	1	-	-	X	-	-	[100,105]
Fumane	Italy	No	A5-A6	40–45	MP-L	Passeriformes	Corvidae	*Pyrrhocorax graculus*	Alpine chough	N/A	Ulna	27	2	-	-	X	-	-	[100]
Fumane	Italy	No	A3-A4	37	M/UP t.—U	Accipitriformes	Accipitridae	*Aquila chrysaetos*	Golden eagle	N/A	Humerus	1	1	-	-	X	-	-	[101]
Gorham`s Cave	Gibraltar	No	IV	38.5–30	MP-M	Accipitriformes	Accipitridae	*Aquila chrysaetos*	Golden eagle	N/A	Ulna	5	1	-	-	X	-	-	[103]
Gorham`s Cave	Gibraltar	No	IV	38.5–30	MP-M	Accipitriformes	Accipitridae	*Gyps melitensis/fulvus*	Griffon vulture	N/A	Femur	14	1	-	-	X	-	-	[103]
Gorham`s Cave	Gibraltar	No	IV	38.5–30	MP-M	Accipitriformes	Accipitridae	*Milvus migrans*	Black kite	N/A	Tibiotarsus	1	1	-	-	X	-	-	[103]
Gorham`s Cave	Gibraltar	No	IV	38.5–30	MP-M	Accipitriformes	Accipitridae	*Milvus milvus*	Red kite	N/A	Radius, Ulna, Coracoid, Cmc	22	4	-	-	X	-	-	[103]
Gorham`s Cave	Gibraltar	No	IV	38.5–30	MP-M	Passeriformes	Corvidae	*Pyrrhocorax graculus*	Alpine chough	N/A	Ulna, Humerus	73	9	-	-	X	-	-	[103]
Gorham`s Cave	Gibraltar	No	IV	38.5–30	MP-M	Passeriformes	Corvidae	*Pyrrhocorax pyrrhocorax*	Red-billed chough	N/A	Ulna, Humerus, Coracoid	180	10	-	-	X	-	-	[103]
Grotte de L’Hyene	France	Yes	n/a	MIS 3	MP	Accipitriformes	Accipitridae	*Aquila chrysaetos*	Golden eagle	N/A	Phalange	n/a	1	-	-	-	X	-	[100,105,107]
Grotte du Noisetier	France	Yes	n/a	MIS 3	MP-D/L	Falconiformes	Falconidae	*Falco sp*.	Falcon	N/A	Humerus	1	1	-	-	X	-	-	[105,108]
Grotte du Renne	France	No	Xb	44–42	C	Anseriformes	Anatidae	*Cygnus cygnus*	Whooper swan	N/A	Ulna	1	1	X	X	-	-	X	[1,109,110]
Grotte du Renne	France	No	IX-X	44–42	C	Accipitriformes	Accipitridae	*Haliaetus albicilla*	White-tailed eagle	N/A	Pedal phalanx	8	2	-	-	-	X	-	[118,119]
Grotte du Renne	France	No	IX-X	44–42	C	Strigiformes	Strigidae	*Bubo bubo*	European eagle-owl	N/A	Pedal phalanx	1	1	-	-	-	X	-	[118,119]
Grotte du Renne	France	No	IX	44–42	C	Accipitriformes	Accipitridae	*Gyps fulvus*	Griffon vulture	N/A	Radius	1	1	-	X	-	-	-	[118,119]
Grotte du Renne	France	No	Xa	44–42	C	Accipitriformes	Accipitridae	*Gyps fulvus*	Griffon vulture	N/A	Radius	1	1	-	X	-	-	-	[118,119]
Grotte du Renne	France	No	Xb	44–42	C	Accipitriformes	Accipitridae	*Gyps fulvus*	Griffon vulture	N/A	Humerus	1	1	-	X	-	-	-	[118,119]
Grotte du Renne	France	No	Xb	44–42	C	Accipitriformes	Accipitridae	*Gyps fulvus*	Griffon vulture	N/A	Humerus	1	1	-	X	-	-	-	[118,119]
Grotte du Renne	France	No	Xc	44–42	C	Accipitriformes	Accipitridae	*Gyps fulvus*	Griffon vulture	N/A	Metaphysis	1	1	-	X	-	-	-	[118,119]
Grotte du Renne	France	No	Xc	44–42	C	indet	indet	indet	indet	eagle or swan	Radius	1	1	X	X	-	-	-	[118,119]
Grotte du Renne	France	No	VIII	44–42	C	Accipitriformes	Accipitridae	*Gyps fulvus*	Griffon vulture	N/A	Ulna	1	1	-	X	-	-	-	[118,119]
Grotte du Renne	France	No	VIII	44–42	C	indet	indet	indet	indet	N/A	diaphysis	1 (8 frg)	1	-	X	-	-	-	[118,119]
Grotte du Renne	France	No	IXb	44–42	C	indet	indet	indet	indet	indet	diaphysis	n/a	n/a	X	X	-	-	-	[118,119]
Grotte du Renne	France	No	IXb	44–42	C	indet	indet	indet	indet	small bird	diaphysis	1	1	-	X	-	-	-	[118,119]
Ibex Cave	Gibraltar	Yes	n/a	37–49	MP-M	Passeriformes	Corvidae	*Pyrrhocorax pyrrhocorax*	Red-billed chough	N/A	Femur, Tarsometatarsus	20	4	-	-	X	-	-	[103]
Krapina	Croatia	Yes	n/a	100–130	MP-M	Accipitriformes	Accipitridae	*Haliaeetus albicilla*	White-tailed eagle	N/A	Phalange	11	5	-	-	-	X	-	[104]
La Ferrassie	France	No	L3a	41–37	M/UP t.—C	Accipitriformes	Accipitridae	*Gypaetus* cf. *barbatus*	Bearded vulture	N/A	n/a	n/a	1?	-	-	X	-	-	[101,111,112]
Lazaret	France	Yes	CII	190–150	MP	Columbiformes	Columbidae	*Columba livia*	Rock dove	N/A	Humerus	12288	1?	-	-	X	-	-	[105,113]
Les Fieux	France	No	Jbase	60–40	MP—MTA	Accipitriformes	Accipitridae	*Haliaeetus albicilla*	White-tailed eagle	N/A	Pedal phalanx	1	1	-	-	-	X	-	[105]
Les Fieux	France	No	I/J	60–40	MP-DM	Accipitriformes	Accipitridae	*Haliaeetus albicilla*	White-tailed eagle	N/A	Pedal phalanx	3	1	-	-	-	X	-	[105]
Les Fieux	France	No	K	MIS 3	MP-DM	Accipitriformes	Accipitridae	*Aquila chrysaetos*	Golden eagle	N/A	Femur	1	1	-	-	-	-	X	[105,114]
Les Fieux	France	No	Ks	MIS 3	MP-MTA	Accipitriformes	Accipitridae	*Haliaeetus albicilla*	White-tailed eagle	N/A	Pedal phalanx	3	1	-	-	-	X	-	[105]
Les Fieux	France	No	Ks	MIS 3	MP-MTA	Accipitriformes	Accipitridae	*Aegypius monachus*	Cinereous vulture	N/A	Pedal phalanx	1	1	-	-	-	X	-	[105]
Les Fieux	France	No	Ks	MIS 3	MP-MTA	Passeriformes	Corvidae	*Corvus corax*	Common raven	N/A	Tibia	21	1	-	-	-	-	X	[105]
Mandrin	France	No	E	52–56	MP-N	Accipitriformes	Accipitridae	*Aquila chrysaetos*	Golden eagle	N/A	Phalange	1	1	-	-	-	X	-	[102]
Pech de l`Aze I	France	Yes	4	58–38	MP-MTA	Accipitriformes	Accipitridae	*Aquila chrysaetos*	Golden eagle	N/A	Pedal phalanx	3	2	-	-	-	X	-	[106,115]
Pech de l`Aze IV	France	Yes	8	100	MP-M	n/a	n/a	n/a	n/a	Medium-sized raptor	Pedal phalanx	1	1	-	-	-	X	-	[116]
Rio Secco	Italy	No	7	48–49	MP-M	Accipitriformes	Accipitridae	*Aquila chrysaetos*	Golden eagle	N/A	Phalange	1	1	-	-	-	X	-	[102]
Salzgitter-Lebenstedt	Germany	Yes	n/a	MIS 3	MP	Anseriformes	Anatidae	*Cygnus sp*.	Swan	N/A	Carpometacarpus	1?	1	-	-	X	-	-	[117]
Salzgitter-Lebenstedt	Germany	Yes	n/a	MIS 3	MP	Anseriformes	Anatidae	*Anas sp*.	Diving duck	N/A	Humerus	1?	1	-	-	X	-	-	[117]
Vanguard	Gibraltar	Yes	n/a	44–50	MP-M	Accipitriformes	Accipitridae	*Gyps fulvus*	Griffon vulture	N/A	Ulna	16	2	-	-	X	-	-	[103]
Vanguard	Gibraltar	Yes	n/a	44–50	MP-M	Passeriformes	Corvidae	*Pyrrhocorax pyrrhocorax*	Red-billed chough	N/A	Humerus	17	1	-	-	X	-	-	[103]
Zaskalnaya VI Kolosovskaya	Crimea	Yes	III	38–43	MP—AK	Passeriformes	Corvidae	*Corvus corax*	Common raven	N/A	Radius	1	1	X	-	-	-	X	[120], This study]

AkK: Ak-Kaya type Micoquian; C: Châtelperronian; CM: Classic Mousterian; Cmc: Carpometacarpus; D: Discoid; DM: Denticulate Mousterian; FE: Feather extraction; frg: Fragments; L: Levallois; MP: Middle Paleolithic; M: Mousterian; MTA: Mousterian of Acheulean Tradition; M/UP t.: Middle/Upper Paleolithic Transition; N: Neronian; n/a: Unknown; N/A: Not applicable; SEMP: Site with exclusively Middle Paleolithic layers; TE: Talon extraction. U: Uluzzian; Sw: sawing;

* calibrated ages.

## Materials and methods

### Archaeological context

**Fig 1:**
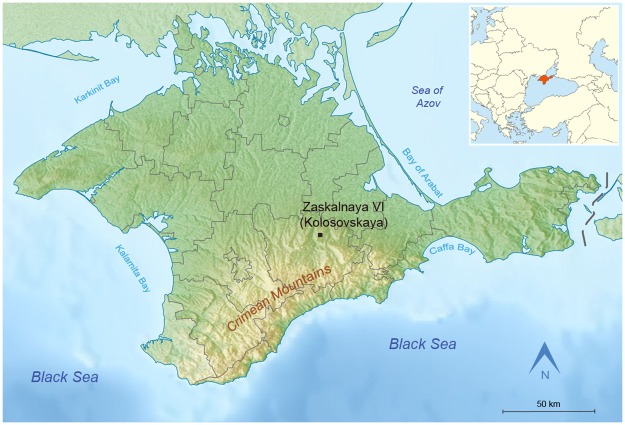
Location of Zaskalnaya VI rock-shelter, Crimea.

**Fig 2:**
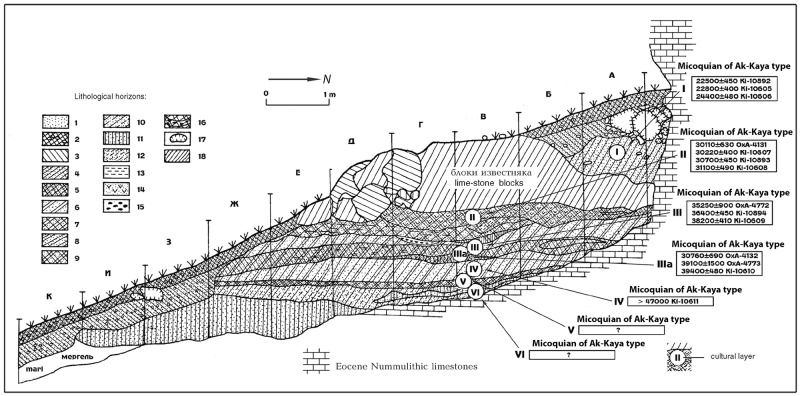
Stratigraphy of Zaskalnaya VI, Crimea. From: [58]–Fig 3.

The Crimean multilayered site of Zaskalnaya VI, also known as Kolosovskaya or the site of Kolosov, after its discoverer Y. G. Kolosov, is located at 45°6' N, 34°36' E, near the village of Vishennoye, Belogorsk District, in the Krasnaya gully, on the right bank of the Biyuk-Karasu river (Fig 1). The shelter has a southern exposure and opens at the foothill zone of the Crimean Mountains, in the eastern part of the peninsula, at an altitude of 205 m above sea level, 60 m above river level, at 35 km distance as the crow flies from the present-day seashore. The height of the rock cliff above the site is 12 m [121,122]. The sedimentary sequence is a collapsed roof above the cultural layer II, which covered a significant part of its ground surface (Fig 2).

Zaskalnaya VI was discovered in 1969 and intensively excavated in 1969–1975, 1977–1978, and 1981–1985 [121–124]. The excavation covered a surface of 78 m^2^, and reached a depth of c. 3 m. In 2005, rescue excavations directed by one of us (VS) was conducted at this site.

The stratigraphic sequence (Fig 2) comprises seven Middle Palaeolithic cultural layers, numbered sequentially by Kolosov and colleagues [121,122] from the bottom to the top (VI-I): lowermost layer VI was excavated over a surface of c.10 m^2^, layer V over an area of 15 m^2^, layer IV over an area of 34 m^2^, layer IIIa, layer III over an area of 42 m^2^, layer II over a surface area of 78 m^2^, and the uppermost layer I over a surface of c.90 m^2^ [121,122].

Between 1969 and 1985 only retouched artifacts, cores, and identifiable large bones were spatially plotted. The remainder of the archeological material was attributed to a cultural layer, a square meter, and the upper and lower depth of the cultural layer in that area of the cave. Sieving with a 2 mm mesh was only occasionally performed. In contrast, all archaeological remains larger than 2 cm from the 2005 excavation were spatially plotted and attributed to a cultural layer. Sediment from this excavation was systematically sieved with a 1 mm mesh. All seven layers of Zaskalnaya VI yielded lithic material described as Micoquian of Ak-Kaya tradition [14,121,125]. No UP layers or isolated UP artifacts were found at the site.

Technologically this industry encompasses both non-Levallois centripetal and sub-parallel non-volumetric core reduction as well as bifacial shaping. Flake tools include points, sidescrapers and backed knives. Bifacial shaping, which reaches up to 30% of the tools, was used to produce foliated points, sidescrapers and the typical ruckenmessers or bifacial back knives.

Fireplaces and pits, including one in layer II containing eight bifaces, were excavated in layers II, III, IIIa and IV. They support the stratigraphic integrity of the site.

The exact chronostratigraphic position of the lowermost layers VI-V has not yet been precisely established, but it is reasonable to assume that they should date to the beginning of the last glacial [126]. Radiocarbon dating of bone samples from the uppermost four cultural layers was performed in the Kiev and Oxford laboratories (Table 2). It indicates that the accumulation of these layers covered a time span ranging from approximately 25 ka to 46 ka. The ages obtained for the layers IIIa, III, II, and I are consistent with those obtained for several other Middle Paleolithic sites from Crimea [14,121,127] and support the hypothesis that this region was a Neanderthal refugium [14,58].

**Table 2:** Radiometric dating of Zaskalnaya VI (Kolosovskaya).

Layer	Material	Lab #	14C Age	Cal BP (95.4%)*	Reference
I	bone	Ki-10892	22500±450	27584–25958	[128]
I	bone	Ki-10605	22800±400	27731–26221	[128]
I	bone	Ki-10606	24400±480	29520–27645	[128]
I	bone	Ki-13373	25700±160	30421–29416	[14]
I	bone	Ki-13375	25200±160	29648–28834	[14]
I	bone	Ki-13376	24600±170	29022–28231	[14]
II	bone	OxA-4131	30110±630	35510–33004	[129,130]
II	bone	Ki-10607	30220±400	34965–33647	[128]
II	bone	Ki-10893	30700±450	35585–33909	[128]
II	bone	Ki-10608	31100±490	36074–34165	[128]
III	bone	OxA-4772	35250±900	41846–38175	[129,130]
III	bone	Ki-10894	36400±450	41847–40127	[128]
III	bone	Ki-10609	38200±410	42936–41820	[128]
IIIa	bone	OxA-4132	30760±690	36243–33666	[129,130]
IIIa	bone	OxA-4773	39100±1500	46566–40973	[129,130]
IIIa	bone	Ki-10610	39400±480	44100–42485	[128]
IV	bone	Ki-10611	> 47000	n/a	[128]

* 14C dates were calibrated with OxCal 4.2 online software using the IntCal 13 curve.

Anthropological remains attributed to Neanderthals have been discovered in layers IIIa, III, and II. They are abundant in layers IIIa and III. Two fragmented mandibles (Zsk VI-72 and Zsk VI-78), fourteen isolated teeth, isolated hand phalanges, a fragmented arm, a forearm bone, and a shin bone were recovered in layer III. Remains of three juvenile Neanderthals, possibly associated with a burial pit, and representing the remnants of a triple burial, come from layer IIIa. A fragmented mandible Zsk VI-72 (left half and a fragment of the right half) with three teeth, along with fourteen isolated teeth, and isolated hand phalanges were recovered in an area of approximately 40 cm of diameter at the limit between squares 32D and 32E. A bifacial tool [58,121] was found in close proximity to the mandible fragment Zsk VI-72. The analysis of the anthropological material showed that the remains belong to two adolescents, a mandible fragment to an individual with the estimated age of 10–12 years, and hand phalanges to an individual aged 14–15 years [58,131–134]. A fragmented right half of mandible Zsk VI-78 and four isolated teeth associated with it were recovered in square 35G, along with fragmented radius, left humerus, and right tibia. These remains belong to two adolescents, the mandible fragment to an individual of 14–15 years, the fragmented humerus bone to an individual of 10–12 years [131,132]. Despite the absence of a burial pit, some have suggested that these remains could be interpreted as two reworked child burials [61,122].

**Fig 3:**
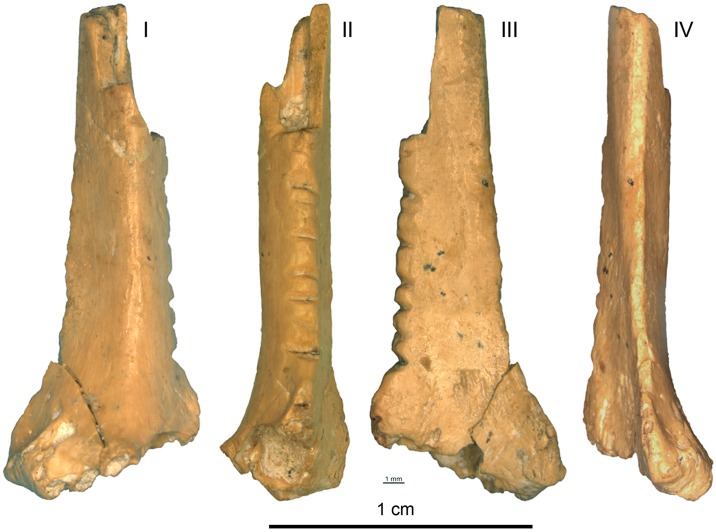
A *Corvus corax* bone fragment with notches from Zaskalnaya VI, layer III. Scale = 1 cm.

The artifact analysed in this paper (Fig 3) was recovered among the remains of avifauna from layer III and described by Tsvelykh and Stepanchuk as an intentionally notched object, possibly used as an eyeless needle in which the notches may have been used to fix a thread and as decoration [120].

### Layer III

Identified all over the site surface and c. 20–30 cm thick, this layer is composed of yellow loamy soil mixed with gravel (Fig 2).

Lithics from layer III are mainly made of fine-grained gray Cretaceous flint, rarely from black and semitransparent brown-colored flint [121]. The majority of artifacts from layer III are not patinated, and contrary to those from layer II, are not covered by concretions [58]. Cores from this layer are flat unifacial (11), bifacial (8) centripetal, and subparallel. Tools include simple (249), double (43), convergent (70) and canted (122) sidescrapers, flake points (36), knives (174), and denticulates (17), other tool types being rare. Bifacial tools are represented by spearheads (4), points (6), scrapers (49), and back knives (111). The scarcity of pre-cores (16) and bifacial forms, the comparatively small size of the cores, and the relatively high frequency of exhausted post-cores and fragmented tools, indicates an intensive use of the lithic raw material [58,121,122].

The fauna from layer III includes mammoth, rhinoceros, horse, saiga, megaloceros, reindeer, red deer, wolf, hare, and small rodents (Table 3). Marine mammals are represented by the remains of the Black sea (short-beaked) common dolphin.

**Table 3:** Faunal remains from Zaskalnaya VI (Kolosovskaya), layer III.

Species	NISP	MNI	Reference	NISP	MNI	Reference
*Mammutus primigenius*	110	4	[122]	317	2	[58]
*Coelodonta antiquitatis*	-	-	-	4	2	[58]
*Equus caballus*	33	4	[122]	33	3	[58]
*Saiga tatarica*	69	6	[122]	54	1	[58]
*Megaloceros giganteus*	2	2	[122]	-	-	-
*Cervus elaphus*	-	-	-	1	1	[58]
*Rangifer tarandus*	1	1	[122]	5	1	[58]
*Canis lupus*	-	-	-	4	1	[58]
*Delfinus delphinus ponticus*	-	-	-	2	1	[58]
*Lepus sp*.	1	1	[122]	-	-	-
*Rodentia indet*.	6	-	[122]	-	-	-
Indetermined	-	-	[122]	6525	-	[58]

Faunal remains are heavily fragmented. Although modifications by medium size carnivores are observed, humans are identified as the main agent of bone accumulation and modification [135]. Mammoth bones appear to have been intentionally collected to be used as fuel [135]. Some mammoth bones display long-term weathering and low content of organic matter; others have a relatively fresh appearance, show flake scars, impacts from use as retouchers, or appear to have been shaped into wedge-like artifacts. Horse limb bones were also used as a raw material for artifact production. Most bone retouchers and possible bone polishers from layer III (45) were made of horse limb bones [14,122,136].

The presence of burnt bone-rich hearths, the large number of faunal remains, the intense use of lithic material, and the long-distance transport of exotic resources (e.g. tail vertebrae of a young dolphin) suggest that layer III of Zaskalnaya VI reflects long-term occupations [58,121]. The object analyzed here comes from layer III, squares 29–33 Zh and 29–33 E and Z. It was identified in 2013 during the analysis of the faunal remains from the 1974 excavation.

### Bird bone from layer III

The object analyzed in this study is kept at the Institute of Archaeology of NASU, Kiev, Ukraine (specimen designation/number: ZVI/III:011/015.14). As common for bone remains from the layer III, it was covered by a thin layer of concretion that was carefully removed. The skeletal element and species identification is proposed, as for the other bird remains from cultural layer III and adjacent layers II and IV of Zaskalnaya VI, on the basis of the fragment’s diagnostic anatomical features, and comparison with the bird bones from the osteological reference collection of the Paleontological Museum of the Central National Natural History Museum of NASU, Ukraine [137]. Descriptive terms of the anatomical elements follow the currently accepted nomenclature used in the analysis of bird remains [138].

Metric data on the archaeological and experimental objects were acquired with a digital caliper. High quality images of four aspects of the archaeological object, and macrophotographs of areas of interest were taken using a NIKON D5300 and a Canon PowerShot S100 digital cameras. Digital images were edited in the Adobe^®^ Photoshop^®^ CS5.1 Extended software. The object was examined with Leica Z6 APOA motorized microscope equipped with a DFC420 digital camera in order to identify and photographically document natural and anthropogenic modifications. Images were treated with Leica Application Suite (LAS) equipped with the Multifocus module, and Leica Map DCM 3D software. The Multifocus module permits the acquisition of extended depth of field images by relying on the adapted algorithms that combine digital images collected at different heights into a single, sharp, composite image. The obtained microscopic images were digitized and edited in the Adobe^®^ Photoshop^®^ CS5.1 Extended software. The Leica Map DCM 3D allowed production of 3D reconstructions of areas of interest. This equipment was also used to measure distances between adjacent notches. Variables recorded for each notch included maximum length, width, depth, and angle, and top, middle, and bottom distance between adjacent notches. Identification of the origin of the bone modifications is based on the experimental reproduction and microscopic analysis of sequential marks produced on bone and stone objects with different tools and motions [139–144].

### Experimental notching

**Fig 4:**
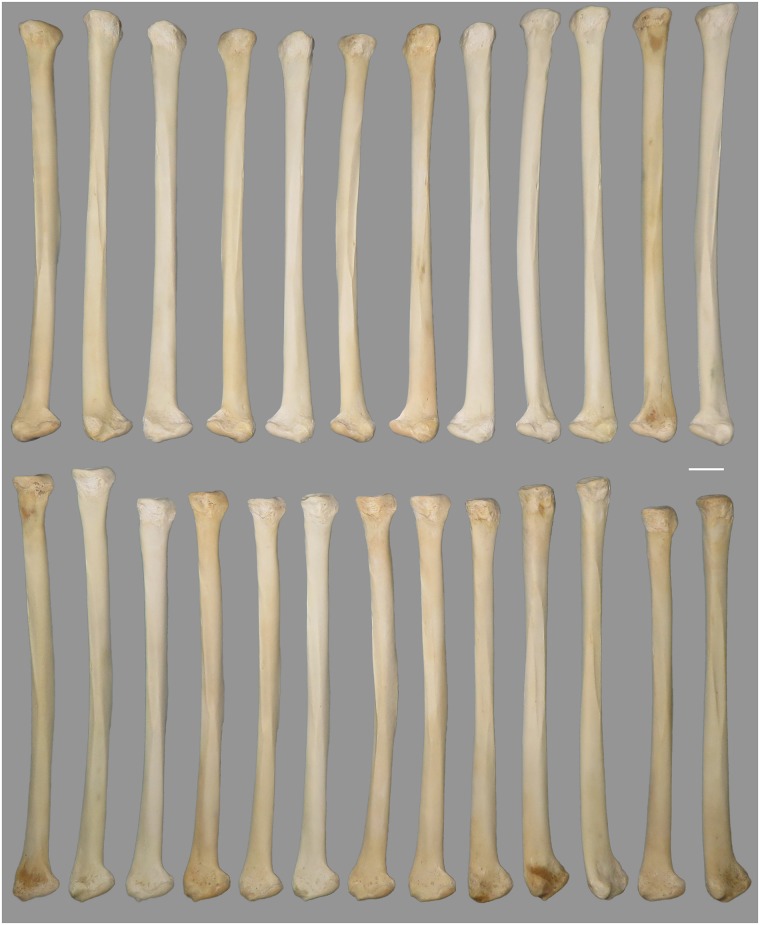
Radii of domestic turkey (*Meleagris ocellata*) used for the experimental phases 3 and 4. Scale = 1 cm.

The experiment was conducted at the PACEA laboratory, Bordeaux University, and involved nine adult subjects, eight right handed and one left handed, seven females and two males (Table 4). The participants provided their informed consent to participate in this experiment. It included four phases. In the first phase, the subjects were asked to use unretouched flint laminar flakes to produce notches by a to-and-fro motion on humeri of domestic turkey (*Meleagris ocellata*). Each subject was given a bone and a tool. The tool was replaced if considered inadequate for the task by the subject. This phase of the experiment lasted for 15 minutes. In this phase, which had an objective to prepare and introduce subjects to the task, they were not given any further instructions aside of making the notches with the tool provided on a given media. In the second, third, and fourth phase of the experiment the subjects were given precise instructions regarding the task in order to achieve consistency of the results allowing comparison with the archaeological specimen. In the second phase of the experiment, the subjects were asked to produce 14 similar, parallel, and equidistant notches on domestic turkey`s radii bone. This phase lasted for 15 minutes. In the third phase of the experiment, the subjects were instructed to produce seven evenly spaced, parallel, and similar notches on radii of domestic turkey (Fig 4). The radii were selected in order to be of a size similar to that of the archaeological specimen. The precise location within which the notches are present on the archaeological specimen was marked on each experimental radius with two thin lines and the subjects were asked to locate the seven notches in that space, with the first and last notch coinciding with each line. No time restriction was given to accomplish this task. The fourth phase of the experiment was identical to the third. For each of these phases, top, middle, and bottom distances between adjacent notches have been measured as well as the length and width of each notch, and the angle formed by each notch with the horizontal plan.

**Table 4:** Information on gender, age and laterality of the experiment`s subjects.

Subject	Gender	Age	RH	LH
1	F	27	x	
2	F	28	x	
3	M	45	x	
4	F	33	x	
5	F	23	x	
6	F	24	x	
7	F	23	x	
8	F	30	x	
9	M	36		x

F: Female; LH: Left-handed; M: Male; RH: Right-handed.

Metric data on notches produced during the first and second phase of the experiment were acquired with the ImageJ software on images taken with NIKON D200 camera. Metric data on notches produced during the third and fourth phase were collected with the ImageJ software on microscopic images obtained with the same equipment used to analyze the archaeological specimen. In order to evaluate the regularity of the spacing, the Weber fraction was used, allowing us to see if the actual distances between experimental notches fit the pattern predicted by the Weber law. Coefficients of variations (CV) were also calculated for the other measured variables.

### Archaeological comparative sample

Distances between notches were measured on thirty-six linear series of notches cut on seventeen bones [99] from seven Magdalenian sites in Western Europe. The objects selected for this study were thin and cylindrical, semi-cylindrical or tube-like in nature, predominantly represented by bird bones, and yielded one or more series of transverse notches produced along a portion of the bone length. A series was considered the collection of notches in a linear manner along one side of the object, that contained at least three notches such that the distances between them could be compared. General microscopic analysis of the comparative notch morphology suggests that each series was produced in a single session, demonstrating the same overall shape, cutting style, and end-point morphology. In two cases, it was determined that the object was rotated 180° during the production of a series.

## Results

### Zaskalnaya VI avifaunal remains

The remains of 41 bird species have been identified at Mousterian sites [145–149], and 93 species at UP sites [145] throughout Crimea. These species represent one-third of the present day Crimean avifauna [137].

Layer III differs from the other layers of Zaskalnaya VI for the relatively high number of bird remains. Five species were identified in this layer (Table 5), and two species in layers I and II. Zaskalnaya VI is the only Middle Palaeolithic site from Crimea in which three of these species (pheasant, gray heron and garganey) are found. Most of the bird species are represented in the modern-day Crimean fauna [150]. Numerous additional bird remains are present in the form of indefinable fragments.

**Table 5:** The bird bone remains from Zaskalnaya VI.

Species		N	Site	Layer	Square	Skeletal Element			Ref.
Scientific name	Common name					Bone	Part	Side	
*Perdix perdix*	Partridge	5	ZSK VI	1	28 A	Coracoideum	Distal	Left	*
						Sternum	-	-	
						Furcula	-	-	
						Ulna	Proximal	Left	
						Tibiotarsus	Proximal	Left	
Aves indet.		2	ZSK VI	1	28 A	-	-	-	*
*Anas querquedula*	Garganey	1	ZSK VI	2	28 A	Femur	Proximal	Left	*
*Phasianus colchicus*	Pheasant	1	ZSK VI	2	-	Femur	Proximal	Left	*
*Lullula arborea*	Wood Lark	1	ZSK VI	2	-	Humerus	Distal	-	[145]
*Falconiformes* indet.	-	1	ZSK VI	2	-	-	-	-	[147]
*Ardea cinerea*	Grey Heron	1	ZSK VI	3	-	Ulna	Proximal	Left	*
*Aquila chrysaёtos*	Golden Eagle	1	ZSK VI	3	-	Last Phalange	-	Right	*
*Turdus merula*	Blackbird	1	ZSK VI	3	29 B	Humerus	-	Right	*
*Corvus corax*	Raven	1	ZSK VI	3	-	Radius	Proximal	Right	*
*P*. *pyrrhocorax / graculus*	Chough / Alpine Chough	1	ZSK VI	3	29 B	Tarsometatarsus	Proximal	Left	*
Aves indet.	-	1	ZSK VI	3	-	-	-	-	*
Nonpasseriformes indet.	-	2	ZSK VI	4	-	-	-	-	*

ZSK: Zaskalnaya; Ref: Reference;

*This study.

Almost all bird bones from Zaskalnaya VI display evidence of crushing of the epiphyses. No detailed analysis, as performed recently on a number of European and African assemblages [151–153], has been conducted to establish the agent responsible for the damage. A small quantity of bird remains show etched surfaces suggesting that they were originally incorporated in pellets of birds of prey [137]. No clear cutmarks were identified so far on the avifauna.

### The Zaskalnaya VI notched bone

The object (18.14 mm long, 7.06 mm wide, and 2.49 mm thick) is the distal fragment of a right radius (Fig 3). The thin and compact wall of the shaft, the smooth texture of the outer surface and the slightly angular shape of the distal end, indicate that it belongs to a bird rather than a mammal of comparable size. The morphology of the epiphysis and the shaft—in particular the marked ligamental prominence—and the greatest breadth of the distal end (Bd) point to the common raven (*Corvus corax*) as, by far, the more probable species [154].

**Fig 5:**
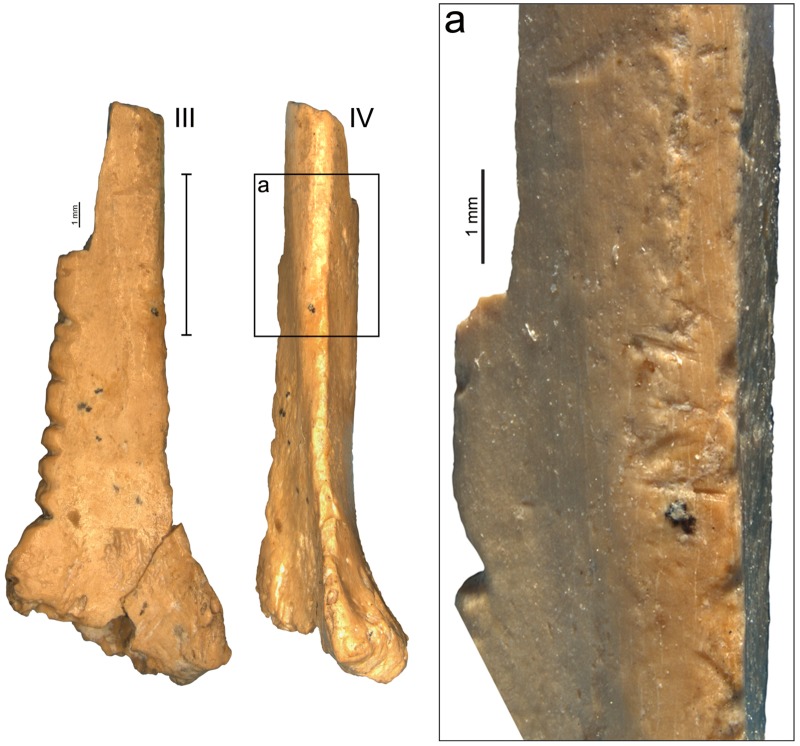
Cutmarks on the anterior edge of the radius bone fragment, Zaskalnaya VI, layer III. a. Localization of the cut marks. Scale = 1 mm.

Its medullary cavity is infilled with hardened sediment (Fig 3 –aspect II). The fragment was partly covered by concretions before cleaning and a microcrack on the distal end of the bone is still covered at places by micro-concretions (Fig 3 –aspect I). Black spots, probably of manganese, are present on the periosteal and medullar surface, and on the breakage. The bone displays the same color, patina and comparable state of preservation all over its surface, including the breakage, suggesting the latter is ancient. A group of subparallel cutmarks, slightly oblique to the bone`s main axis, are present on the anterior edge, 1 cm above the styloid process (Fig 5).

### Technological analysis

**Fig 6:**
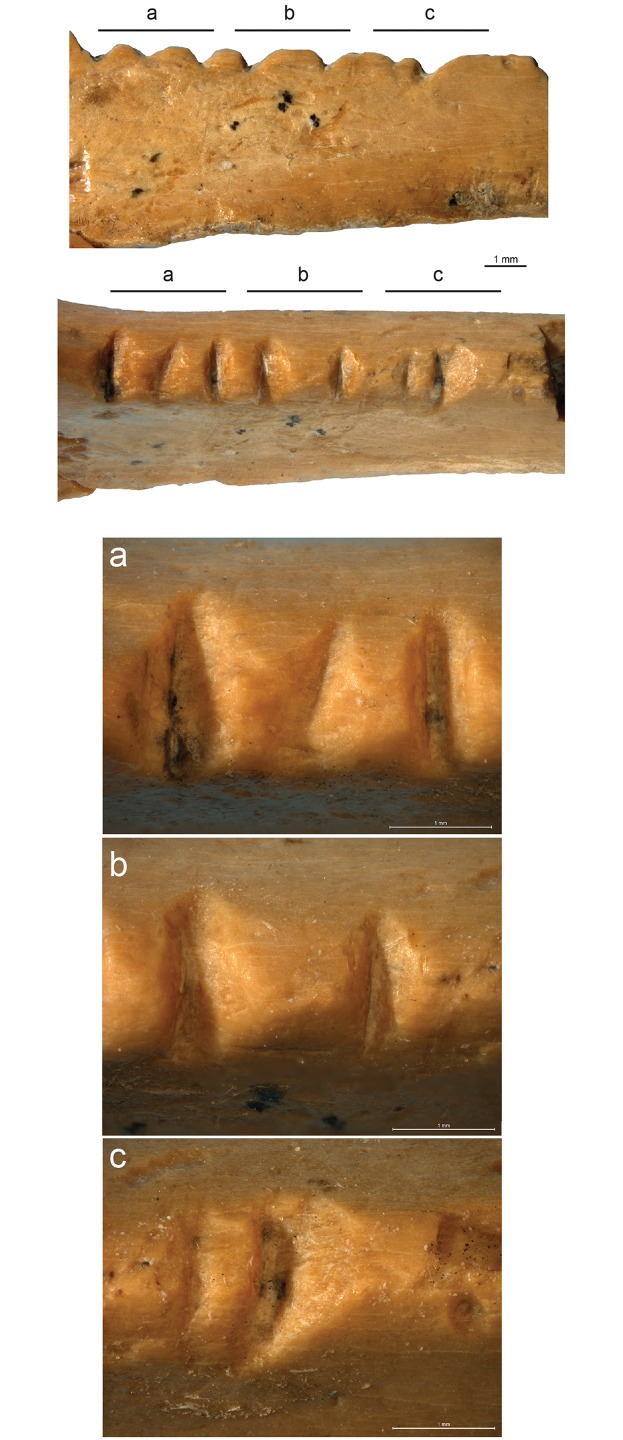
Microscopic images of the notches on the Zaskalnaya VI bird bone fragment from layer III. Profile (top) and en face (bottom) views. Scale = 1 mm. **a**. Magnification of the notches 1–3; **b**. Magnification of the notches 4–5; **c**. Magnification of the notches 6–7.

Seven notches, cut on the posterior aspect of the bone between the epiphysis and the breakage, numbered henceforth 1–7 from the one closest to the epiphysis forward, run over a length of 10 mm (Figs 3 and 6). The surface of each notch is smoothed but still displays microscopic features that allow a technological and metrical analysis (Table 6). Only notch 6 is too superficial to reliably measure the angle formed by the notch walls.

**Table 6:** Morphological and metric data on Zaskalnaya VI notches.

Notch	Length	Max Width	Depth	Orientation	Angle	Section	Bottom	Microsteps
	(mm)	(mm)	(mm)	(°)	(°)			
1	1.758	0.865	0.326	93.252	97	v-shaped	ce	left
2	1.47	0.607	0.220	111.01	115	v-shaped	no	no
3	1.597	0.658	0.285	91.432	90	v-shaped	ce	left & right
4	1.609	0.759	0.337	90.712	98	v-shaped	ce	no
5	1.256	0.678	0.279	95.484	102	v-shaped	ce	no
6	1.124	0.601	0.229	96.621	na	v-shaped	no	no
7	1.471	1.011	0.326	81.815	101	v-shaped	ce	no

ce: closer to the epiphysis

**Fig 7:**
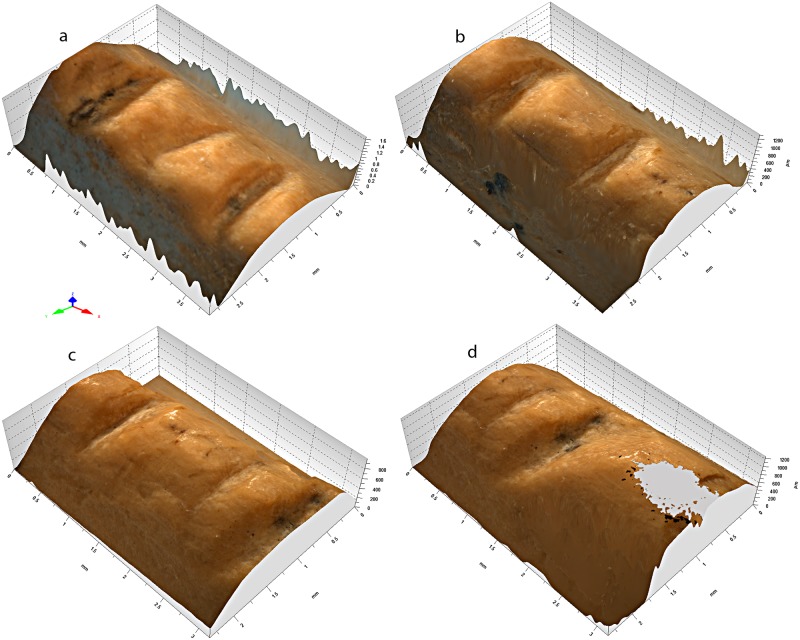
3D reconstruction of the Zaskalnaya VI bone notches. **a**. Notches 1–3; **b**. Notches 4–5; **c**. Notches 5–7; **d**. Notches 6–7.

**Fig 8:**
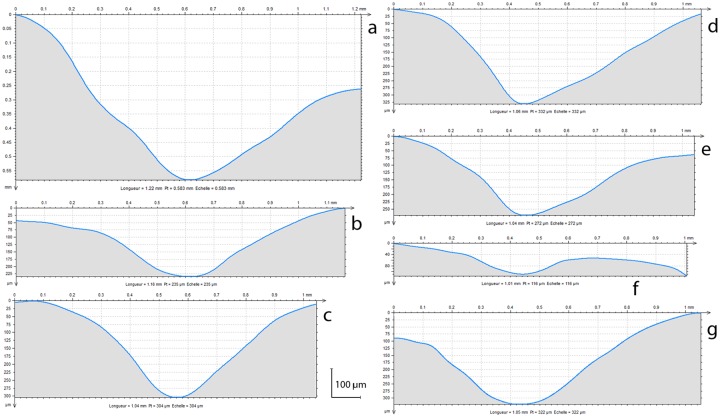
Sections of the Zaskalnaya VI notches. Labels a-g correspond in order to the notches 1–7.

Notches 1, 3–5, and 7 differ from notches 2 and 6 (Figs 6, 7 and 8). The former are parallel and perpendicular to the bone main axis. They are deeply cut and bear striations on the notch bottom indicating that they were produced by the to-and-fro movement of a lithic cutting edge. They all display a comparable asymmetrical section, deeper toward the epiphysis of the bone. On notches cut by the to-and-fro motion of a lithic blade or flake the asymmetry of the notch indicates the location of the ventral aspect of the blank during the cutting process [139]. In the case of notches 1, 3–5, and 7, the fact that the notch wall closer to the epiphysis is steeper indicates that the ventral face of the tool was oriented toward the distal epiphysis of the radius. Similarity in section morphology and angle formed by the walls of these notches, ranging between 90° and 102°, suggests that these notches were made by the same tool in a single session. Gradual increase in the notches’ angle from the epiphysis toward the diaphysis suggests that that the craftsman incised notch 1 first and juxtaposed notches 3–5, and 7 toward the middle of the diaphysis. The type of tool used can be inferred from the notch angles and morphology. Retouched cutting edges generally produce asymmetrical notches with a flat steep side, corresponding to the ventral side of the blank, and a more oblique side displaying multiple steps parallel to the notch bottom, due to the action of the retouch. Sections of notches produced by retouched tools form angles ranging between 60° and 95°. Unretouched cutting edges produce more symmetrical notches with flat sides and angles ranging between 35° and 65°. Angles of notches 1, 3–5, and 7 are wide and fall at the very limit of the notches produced by retouched cutting edges. However, they do not bear the multiple steps on the wide side typical of notches made by retouched tools. Notch 1 presents a single step on the steep left side and notch 3 a step on both sides. No steps are recorded on the others. This evidence, together with a relatively high degree of variability in the overall morphology of these notches, is consistent with the use of a very robust unretouched flake.

Notches 2 and 6 are parallel and oriented obliquely to the bone's main axis. They are superficial, have wider and more symmetrical sections than the previous notches, and show irregular edges indicating that they were probably produced by a single passage of a cutting edge. Their different orientation, morphology and production technique indicate that they were probably added after the first set, either with the same tool—perhaps with a different area of the same cutting edge—or a different tool. Although wider than angles measured on notches 1, 3–5, and 7, the angle measured on notch 2 is compatible with the use of the same cutting edge considering that the smoothing affecting the notches may have slightly flattened its surface and that the cutting edge would have been already worn by the production of the previous set of notches when this and notch 6 were incised. Superficial incisions are occasionally produced by mistake when incising notches by a to-and-fro movement of a cutting edge. It is, however, unlikely that notches 2 and 6 result from that process. They are deeper than unintentional notches reported in the literature [139] and observed in our experimental collection. Most importantly side notches produced unwillingly generally display the same orientation of the closest main notch, being the result of the same repeated motion, which is not the case with notches 2 and 6.

### Experimental results

**Fig 9:**
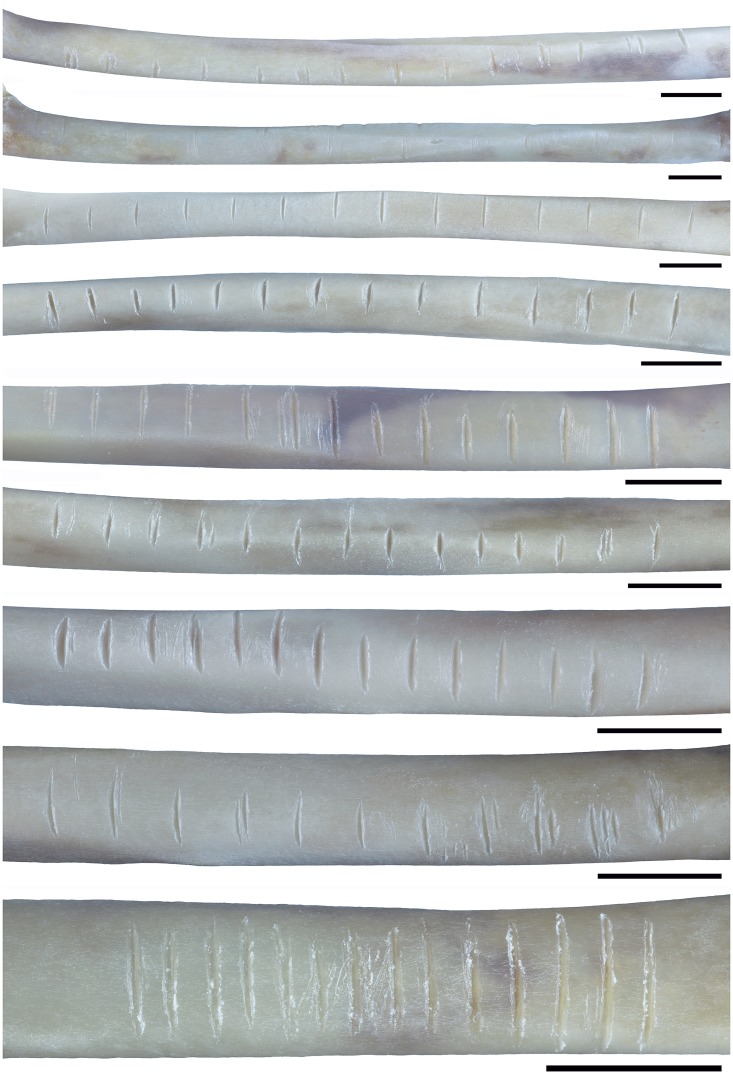
Fourteen notches produced by the modern subjects on turkey`s radii bone during the second phase of the experiment. Scale = 1 cm.

**Fig 10:**
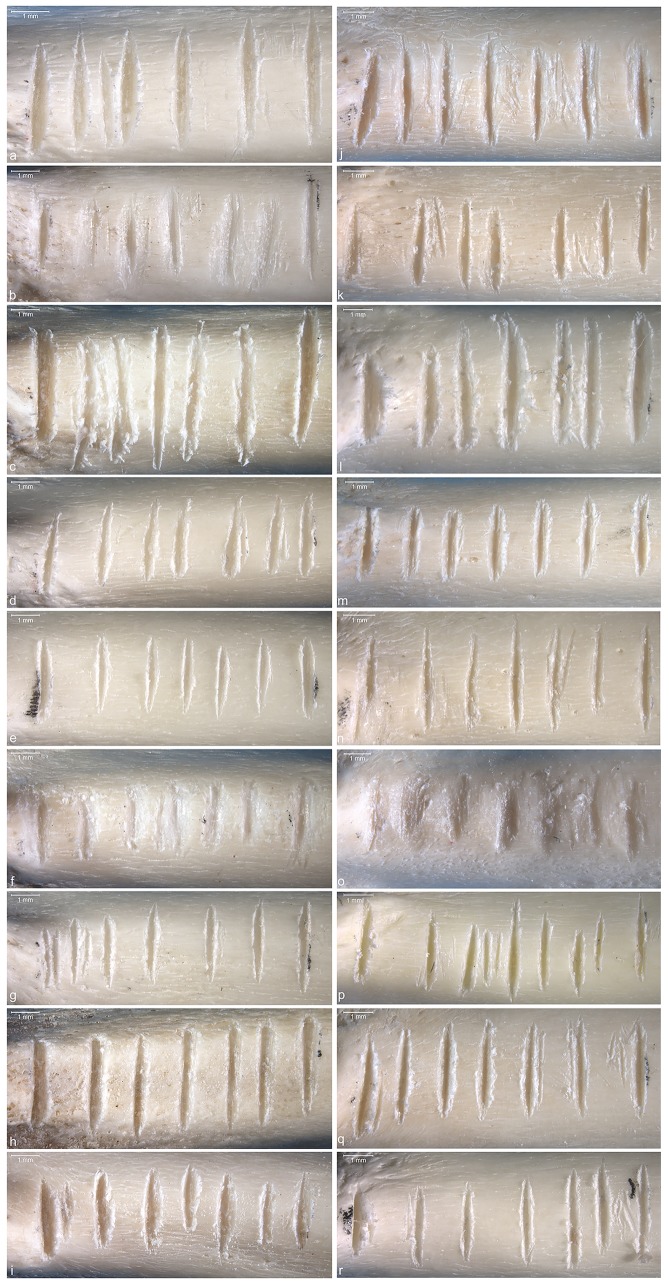
Seven notches produced on radii of domestic turkey during the third (a-i) and fourth (j-r) phase of the experiment. Aligned are the sets made by the same subjects.

During the first phase of the experiment nine subjects incised in total 129 notches on nine domestic turkey’s humeri bones. They produced between 6 and 41 notches per bone, with the average of 14.3 notches per bone. Two subjects cut 6 notches, two other 13 notches per bone. Five subjects produced 9, 11, 14, 16 and 41 notches per bone. The lack of constraints in this phase of the experiment is reflected by discrepancy in placing, distance and orientation of the notches. Some subjects produced notches in continuity on one aspect of the bone, others on different aspects. The length, width, orientation, placing, and distance between notches were highly variable. During the second phase of the experiment, a single subject produced 12 instead of 14 notches, while others fulfilled the task in terms of number of notches required, making in total 124 notches (Fig 9). Therefore, aside from a single mentioned exception for which 11 gap measurements were gathered, 13 measurements of distances between top, middle, and bottom of adjacent notches have been obtained for each specimen, constituting a total of 345 individual measurements (experimental set C). During the third and fourth phase of the experiment, all subjects conformed to the given constrains, producing in total 63 notches (Fig 10). As done for the previous phase, distances between top, middle, and bottom of adjacent notches have been measured for each specimen, comprising 54 measurements per each category (top, middle, and bottom distances), and a total of 162 individual measurements taken together—experimental sets A and B.

#### A Weber-Fechner law for notches on bone rods

The constant represented by the Weber Fraction (0.029–0.030, or a JND of 3%) was originally established on planar surfaces [92, for a detailed discussion see 99], yet it must be acknowledged that the lengths in question here are of a three-dimensional nature on variably curved surfaces. More recent psychophysical experiments have demonstrated that humans find it more difficult to evaluate distance on curved surfaces and in three dimensions; the ability to estimate the length of lines on curved surfaces in 3D settings yields an error that varies dramatically from individual to individual and is dependent upon factors such as viewing distance, orientation of the surface and/or line and type of surface [155–157]. Such results are not directly applicable to the results of our experiments and the analysis of archaeological series as we wish to evaluate the *difference* threshold between distances rather than the perception of absolute length. Nevertheless, it is plausible to consider that the ability to discriminate distances between notches will be influenced by their existence upon a curved surface and, thus provide a wide-ranging and non-constant error. Furthermore, the notches under examination are not only produced on surfaces of varying curvature, but a production error will be incurred with respect to the materials and practices being used to produce a notch on a bone surface. Whilst individuals may be able to *perceive* exactly where they wish to place the notch on the bone, an error may exist due to the nature of the bone surface, flint tool and the individual's ability at producing the notch.

An evaluation of the distance measurements for the experimental series demonstrates that the Weber Fraction of 3% is indeed met very rarely and seems to be rather an exception than a rule. In actuality, the experimental production of equidistant sets of notches, submitted to the above biases, can be used to evaluate the Weber Fraction or difference threshold specific to the production and perception of notches made by adult modern humans on bone rods with a to-and-fro movement of a lithic cutting edge, with the intention of making them similar in length, parallel and equidistant. In addition, two of our experiments incorporated constraints of space that allow us to evaluate to what extent this factor may influence the production of sequential notches and make results particularly valuable to assess the archaeological set of notches under study.

**Fig 11:**
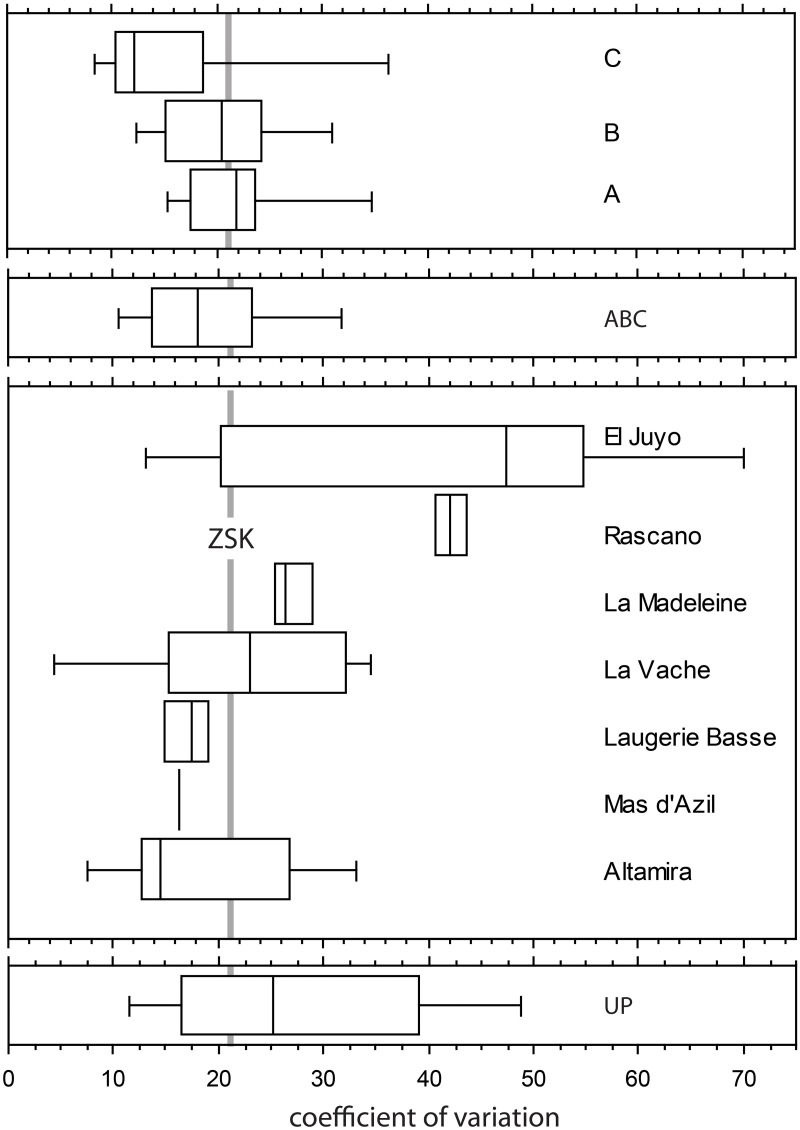
Boxplots representing the coefficient of variation for distances of the experimental and archaeological sets of notches.

The coefficient of variation for distances has been calculated for the three experiments, summarized in Table 7 and Fig 11, and is compared to the value obtained for Zaskalnaya set. Most CVs calculated on sets produced during the unconstrained experiment are lower than 20% (mean = 16%). Most of those from sets made under space constraints range between 15% and 25%, with means of 22% and 21%.

**Table 7:** Coefficients of variation for distances of the experimental and archaeological sets of notches.

	Series of notches	Mean	SD	Minimum	Maximum
Experimental set A	9	22.345	7.9	14.722	41.423
Experimental set B	9	20.639	6.907	11.004	32.332
Experimental set C	9	16.729	11.517	7.057	44.746
Experimental Total	27	19.904	8.968	7.057	44.746
Cueva del Juyo	9	41.722	21.515	10.4	72.1
Cueva del Rascaño	2	42.15	2.192	40.6	43.7
Abri de la Madeleine	4	27.175	2.644	25.1	31
Grotte de La Vache	6	21.917	11.886	3.2	34.9
Abri de Laugerie Basse	4	17	3.369	12.4	20.5
Grotte du Mas d'Azil	1	16.3	n/a	16.3	16.3
Cueva de Altamira	5	18.9	10.056	7.6	33.2
UP Total	31	26.452	8.61	3.2	72.1
Zaskalnaya VI	1	20.531	n/a	20.531	20.531
Zaskalnaya VI—5 notches	1	30.75	n/a	30.75	30.75

Analysis of UP notched bird bones [99] identified only a single series of notches with a coefficient of variation close to the Weber Fraction for patterns on planar surfaces (0.032 or 3.2%). The overall distribution of the coefficient of variation for the distance between two notches highlighted two populations, the first of which fell predominantly between 10% and 25%, and peaked between 15% and 20%. This population, consisting of 15 series, is interpreted to be composed of cases in which the notches were perceived as regularly spaced.

The second population yielded a coefficient of variation largely between 30% and 55%, with an upper limit case of 0.721 (72.1%). This population was posited to represent cases for which the spatial distribution of notches were perceived to be unequal or random, and was considered to include a further 16 examples [99].

The remaining five series of the archaeological sample from the UP represent examples that cannot be considered as regular or random in their spatial distribution but rather display a particular trend in their layout, and should thus be acknowledged. While they demonstrate wide-ranging values for the CV, that would have indicated them as either regularly or randomly distributed, the distances between notches were shown to either increase or decrease, to some extent, over the course of the series. These examples included the two cases in which the bone was rotated 180° during the production of the series. Furthermore, a third example, with a total of 18 notches, included the first 10 notches being produced at distances which would be interpreted as regularly spaced (with a CV of 0.12 or 12.0%) and the remaining 8 notches being increasingly spread out in a linear manner. This case demonstrates a mixture of regular and increasingly distributed notches. It is important, therefore, in the context of this study, to acknowledge that a random spatial distribution is not the only alternative to regularly spaced notches, but that they can also demonstrate alternative and mixed trends in their spatial distribution that would not be reflected by their coefficient of variation.

The CV of distances between the notches on the ZSK specimen is 20.5%, a value that falls within the range of variation for regularly spaced experimental and UP sets of notches and is almost identical or very close to the mean CV calculated on experimental sets of notches cut on the same space available to the Neanderthal craftsman. However, the CV of the ZSK set of notches would have been 30.7%, if notches 2 and 6 were not added.

#### Multivariate analysis

**Fig 12:**
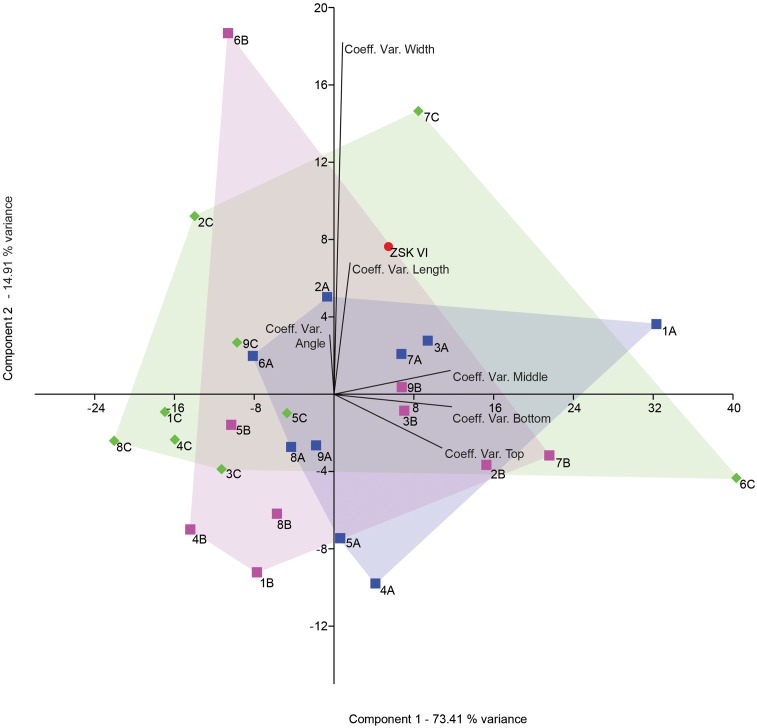
PCA scatter diagram showing distribution of CV for experimental specimens from the phases 2 (1C-9C), 3 (1A-9A) and 4 (1B-9B), and the archaeological object. The coefficient of variation was calculated for the following six variables: distances top, distances middle, distances bottom, length, width, and angle.

The first two components of a PCA using the CVs of the seven morphometric variables recorded on the ZSK notches and series of notches from experimental phases two, three and four account for 90.6% of the variance (Fig 12). They identify the main factors underpinning the differences between these sets. Component 1, mostly influenced by distances between notches, indicates that series from experiment 2 feature the highest variability but also a consistent number of series showing the more regularly spaced and aligned notches. Comparatively, series of notches belonging to experiment 3 and 4 are less regularly spaced and aligned than those from experiment 2. This is likely due to the space constraints imposed during these experiments. Series from experiment 4 split into two groups, one composed of more and the other less regularly spaced notches than those from experiment 3. The ZSK notches fall in the very middle of the experiment 3 and 4 variability. Component 2, mostly influenced by the notches’ width and, to a lesser extent, length and orientation, reveals that most of the series of notches from experiments 3 and 4 are characterised by a lower or similar variability when compared to those from experiment 2. This can also be attributed to the imposed space constraints: the engraver is obliged to consider also the notch size if he/she needs to fit a given number of notches in a small space. ZSK notches fall within the range of experiments 2 and 4 but are clearly more variable in term of notch size and orientation than the large majority of the series from the three experiments. This is due, as made clear by the technological analysis of the ZSK notches, to the fact that unlike the experimental series, the alignment of notches on the raven bone consists of an accumulation of two sequences of notches with a significant change in notch orientation and size between them. The higher coefficients of variation generated by this action is responsible for the outlying position of the ZSK set of notches.

## Discussion

The technological analysis of the notches on the ZSK raven bone indicates that two of them (2 and 6) were apparently added to fill in the gap left between notches 1–3 and 5–7. The possibility that the space between these notches was left on purpose, in order to add at a later stage notches of different length and orientation, with the goal of creating a special pattern composed of longer perpendicular and short oblique notches, is unlikely. It would have been easy for the craftsman to alternate during the same session deeper notches with more superficial and obliquely oriented ones. It is more probable that the Neanderthal took the decision of adding notches 2 and 6, after completing the first set and realizing that its production left two gaps. Two reasons may account for this action. The craftsman may have considered that, if made for functional reasons, i.e. to facilitate the grip of the object, the notches of the first set were not frequent and numerous enough to fulfill that function. Alternatively, he/she may have considered, irrespective of the possible functional reason behind the production of the notches, that it was important to add two notches in order to create a visually more regular and consistent pattern. The second hypothesis is in our view the more probable because adding of the two very small and superficial notches added virtually nothing to the gripping power of the object`s surface.

This interpretation is strengthened by the difference between the perception of the notches after their first accumulation and completion. The identification, achieved in the present study, of the Weber fraction for sets of notches made on small rods and its application to the ZSK notches prior to adding notches 2 and 6 demonstrates that the sequence would not have been perceived as regularly spaced by the UP and living modern individuals. The sequence of notches would have been perceived as regularly spaced by those same individuals after adding notches 2 and 6. This conclusion is supported by results of our analysis of a large sample of experimental alignments of notches made under technological and neuromotor constraints similar to those at work when the ZSK Neanderthal craftsman incised the raven bone, with the deliberate intention of producing equidistant notches. It is also consistent with results obtained when applying this approach to more recent archaeological examples of aligned notches. Thus, adding of the two additional notches on the ZSK raven bone appears to be consistent with the intention to make the notches of final series regularly spaced. This suggests that Neanderthals were perceiving and discriminating equidistant from unequally spaced sequential marks in a way similar to us and that their neuromotor control allowed them to master the techniques and motions necessary to obtain regularity when required. Such conclusion remains valid independently of the functions that the ZSK object might have had. The Neanderthal intention, highlighted by our study, of producing notches that can be perceived as equidistant makes it less likely that they were incised on the raven bone for purely functional reasons such as securing grip during the use of the object as an awl or to fix a thread to use it as an eyeless needle. These and other, including symbolic, functions are entirely possible considering the fragmentary state of the object. It is clear that in order to effectively fulfill any such function the object ‘had’ to be incised with notches, but not necessarily equidistant notches in case of a solely utilitarian purpose. Still, the results of the study pinpoint that a clear effort has been put to reach the goal of producing not just random but instead equidistant notches, that would have been perceived as regularly spaced. This implies that the resulting pattern could have conveyed an information, not directly linked to the object function, communicating to the user, and likely other members of the Neanderthal group. In this respect the sequential notches on the ZSK raven bone represent the first case of bird bone use by Neanderthal for which a symbolic function can be argued on direct rather than circumstantial evidence.

The research strategy followed here should be adapted and extended to other Lower and Middle Palaeolithic incised objects in order to establish when the faculty for precisely discriminating regularly from irregularly spaced marks arose and identify the role it may have played in creating symbolic codes.
